# Landscape of stress: Tree mortality influences physiological stress and survival in a native mesocarnivore

**DOI:** 10.1371/journal.pone.0253604

**Published:** 2021-07-01

**Authors:** Jennifer R. Kordosky, Eric M. Gese, Craig M. Thompson, Patricia A. Terletzky, Lorin A. Neuman-Lee, Jon D. Schneiderman, Kathryn L. Purcell, Susannah S. French

**Affiliations:** 1 Department of Wildland Resources, Utah State University, Logan, Utah, United States of America; 2 U.S. Department of Agriculture, Wildlife Services, National Wildlife Research Center, Logan, Utah, United States of America; 3 U.S. Department of Agriculture, Forest Service, Missoula, Montana, United States of America; 4 Department of Biology, Utah State University, Logan, Utah, United States of America; 5 U.S. Department of Agriculture, Forest Service, Pacific Southwest Research Station, Fresno, California, United States of America; Universitat Autonoma de Barcelona, SPAIN

## Abstract

Climate change and anthropogenic modifications to the landscape can have both positive and negative effects on an animal. Linking landscape change to physiological stress and fitness of an animal is a fundamental tenet to be examined in applied ecology. Cortisol is a glucocorticoid hormone that can be used to indicate an animal’s physiological stress response. In the Sierra Nevada Mountains of California, fishers (*Pekania pennanti*) are a threatened mesocarnivore that have been subjected to rapid landscape changes due to anthropogenic modifications and tree mortality related to a 4-year drought. We measured cortisol concentrations in the hair of 64 fishers (41 females, 23 males) captured and radio-collared in the Sierra National Forest, California. We addressed two main questions: (1) Is the physiological stress response of fishers influenced by anthropogenic factors, habitat type, canopy cover, and tree mortality due to drought in their home range? (2) Does the physiological stress response influence survival, reproduction, or body condition? We examined these factors within a fisher home range at 3 scales (30, 60, 95% isopleths). Using model selection, we found that tree mortality was the principle driver influencing stress levels among individual fishers with female and male fishers having increasing cortisol levels in home ranges with increasing tree mortality. Most importantly, we also found a link between physiological stress and demography where female fishers with low cortisol levels had the highest annual survival rate (0.94), whereas females with medium and high cortisol had lower annual survival rates, 0.78 and 0.81, respectively. We found no significant relationships between cortisol levels and body condition, male survival, or litter size. We concluded that tree mortality related to a 4-year drought has created a “landscape of stress” for this small, isolated fisher population.

## Introduction

The “landscape of fear” concept has been postulated to drive the behavioral responses of many species to perceived predation risk that cascades from individuals to ecosystems [1–4]. For example, the reintroduction of wolves (*Canis lupus*) has changed the behavior of elk (*Cervus elaphus*) in Yellowstone National Park with elk shifting their diel activity to using risky places during wolf downtimes [5]. Similarly, introduction of Tasmanian devils (*Sarcophilus harrisii*) to Maria Island, Tasmania, influenced the foraging behavior of their prey, the common brushtail possum (*Trichosurus vulpecula*) with giving-up density being 64% higher than a devil-free site [6]. Even the fear of humans as the apex predator can influence wildlife communities [7]. We hypothesize that a “landscape of stress” also exists when environmental attributes place an animal is a state of chronic stress, and this stress response is subsequently linked to changes in components of fitness. The next challenge for stress research should be relating glucocorticoid secretion and measures of fitness [8].

Physiological stress in wildlife is often measured with cortisol concentrations, typically collected through blood, scat, feathers, and hair [9–12]. Cortisol is a glucocorticoid hormone released to mobilize energy in response to stress [13] and is incorporated in the hair shaft via passive diffusion during periods of active hair growth providing a long-term record (weeks to months) of both baseline physiological activity and stress responses [14]. The relationship between glucocorticoid concentrations measured in blood, hair, and scats, and animal stress is well established [15–18], and has been measured across numerous species, from garter snakes (*Thamnophis sirtalis*) to polar bears (*Ursus maritimus*) [18–20]. Past studies have found animals exposed to constant environmental stressors, such as predator abundance, weather, climate change, or human activity have elevated cortisol levels [12, 21, 22]. These elevated levels can negatively affect reproduction [23–25], increase vulnerability to diseases [26, 27], and reduce immune function [28, 29] causing mortality [30]. Stress can have indirect effects on survival as individuals with decreased fitness may have difficulty persisting in a “landscape of stress” [31].

Climate change and anthropogenic modifications to the landscape can have both positive and negative direct and indirect effects on wildlife [22, 32, 33]. As climate change and other anthropogenic influences impact the landscape, it is especially important to monitor the responses of small populations [34]. Extreme environmental characteristics, such as increased temperature or reduced precipitation, can act as chronic stressors that challenge physiological systems in animals [35, 36] and decrease measures of fitness such as survival and reproductive success [28, 37, 38]. Additionally, anthropogenic modifications to the landscape can influence stress levels for many species. For example, one study documented marine iguanas (*Amblyrhynchus cristatus*) had stress-induced elevations in plasma corticosterone among tourist-exposed populations relative to undisturbed populations [16].

Fishers (*Pekania pennanti*) are mesocarnivores dependent on dense, complex, multi-story forest [39–41]. Historically, fishers in California were distributed throughout the Sierra Nevada; however, due to trapping, habitat loss, and fragmentation, fisher densities are now lower than they were in the 1800s before these disturbances occurred [42–44]. In the 1940s, fisher populations in California declined and fishers were listed as a species of concern; as a result, a trapping ban was instituted in 1946 [45]. Due to the effects of overharvest, habitat loss, and landscape fragmentation, the fisher population in the southern Sierra Nevada has not recovered [43, 44]. In 1990, fishers were petitioned for listing as a threatened species under the Endangered Species Act. Currently, the southern Sierra Nevada population is small (<300 adults [46, 47]) and genetically isolated [47–49]. Factors which were identified as potentially limiting recovery of this fisher population include the influence of climate change (mainly more frequent and longer droughts) and anthropogenic modifications (e.g., logging, development) to the landscape, since trapping has been banned for many years [50].

In the Sierra Nevada Mountains, California, a 4-year drought has affected the forest through mountain pine beetle (*Dendroctonus ponderosae*) infestation and tree mortality, as well as increased fire activity and severity [51]. Climate change will continue to increase the severity and frequency of droughts and insect outbreaks [52, 53], potentially influencing tree health and mortality. Additionally, anthropogenic modifications to the forest, such as continued development, logging, and prescribed burning, created a highly fragmented and constantly changing forest, further influencing the landscape [54, 55] which fishers must contend with when seeking suitable habitat. The increase in human activities in the Sierras led to the question of whether habitat changes associated with anthropogenic modifications and tree mortality related to a 4-year drought is directly or indirectly affecting the native fisher population through increased stress.

Concerns over low population numbers and conflicts with fuel reduction efforts led to the initiation of two studies beginning in 2007 that investigate the ecology and population dynamics of fishers in the Sierra Nevada [40, 41, 56, 57]. From 2007 to 2016, >250 fishers were radio-collared and monitored to determine their habitat use, survival, and reproduction. Results from these studies found that fishers preferred late-successional forests of dense tree cover within multi-layered old growth stands [40, 58], suggesting that altering the forest could negatively influence this isolated population. Our goal was to examine the influence of anthropogenic modifications, habitat, and tree mortality caused by a 4-year drought on the physiological stress response and fitness of Pacific fishers in the Sierra Nevada Mountains. Our objectives were to determine what anthropogenic modifications (silvicultural treatments, road and building densities), habitat (vegetative type, canopy cover), and tree mortality influenced cortisol concentrations among individual fishers. We also examined the relationship between cortisol and measures of fitness (body condition, survival, reproduction) to examine the effects of stress on fitness. We quantified metrics of anthropogenic modifications, habitat, and tree mortality across three home-range use scales (30, 60, 95% isopleths) for fishers in the Sierra Nevada, California. Results from this study can be used to inform forest managers and conservation practitioners, aiming to protect this small, isolated population of fishers.

## Methods

### Ethics statement

Fieldwork was approved by the United States Department of Agriculture, Forest Service, and the United States Department of Agriculture’s National Wildlife Research Center. Permission to access land on the Sierra National Forest was obtained from the United States Forest Service, Bass Lake and High Sierra Ranger Districts.

We captured, immobilized, and handled animals under authorization of the United States Forest Service with permits from the California Department of Fish and Wildlife (Permit SC-2730), and review and approval of capture and handling protocols from the Institutional Animal Care and Use Committee at the University of California-Davis (IACUC #18022).

### Study area

This study was conducted in the Sugar Pine (SP) and Kings River (KR) study areas located in the Sierra Nevada Mountains of central California, USA. The 800-km^2^ SP study area (37°19’29.5"N, 119°31’31.8"W) was located near Oakhurst, California, while the 450-km^2^ KR study area (37°05’45.2"N, 119°14’35.8"W) was located near Shaver Lake. The KR study area was located approximately 11 km southeast of the SP study area. Main habitats in the study areas [59, 60] included conifer forests at higher elevations dominated by ponderosa pine (*Pinus ponderosa*), sugar pine (*P*. *lambertiana*), Jeffery pine (*P*. *jeffreyi*), white fir (*Abes concolor*), and incense cedar (*Calocedrus decurrens*). Lower elevations had hardwood forests dominated by California black oak (*Quercus kelloggii*) and canyon live oak (*Q*. *chrysolepis*) or a combination of conifer and hardwood forest. The understory often consisted of shrubs including manzanita (*Arcostaphylos spp*.), white thorn (*Ceanothus leucodermis*) and bear clover (*Chamaebatia foliolosa*). During summer, precipitation was rare with maximum temperatures averaging 23° C and minimum temperatures averaging 9° C (https://weather.com/weather/monthly/l/Shaver+Lake+CA+93664:4:US). During winter, snow accumulation was typical throughout the Sierras, with both study areas having snow cover from November-April. Winter temperatures averaged a high of 7° C and a low of -4° C. Both areas were similar in elevation from 1,000 to 2,400 m. The region was impacted by a 4-year (2012–2015) drought, resulting in high levels of tree mortality due to a combination of the drought and mountain pine beetle infestation [61].

### Capture and monitoring

Cortisol levels in hair are an accumulation of physiological stress responses over several months, and can therefore be used as a measure of chronic stress [20, 62–66]. Hair hormone concentrations can be used as a minimally invasive alternative to serum concentrations, which must come from animal captures. Hair samples also provide a long-term average measurement of hormone concentrations that is not influenced by short-term fluctuations like that of serum. However, recent evidence has also suggested cortisol may also enter the hair from peripheral sources via glandular secretions or locally from the hair follicle [67, 68]. Therefore, cortisol levels in the hair may represent a combination of chronic and acute timeframes as well as central and peripheral cortisol release [14, 69], but is currently unknown in the fisher. Cortisol concentrations from hair has been shown to be a viable alternative for many animals [14, 70], including several species of mammals [10, 71, 72]. Unfortunately, due to the low numbers of this threatened species, we were unable to perform a physiological validation for this species to directly link stimulation of the hypothalamic-pituitary-adrenal axis to cortisol levels in the hair [69, 73].

During 3 trapping seasons (2014–2015, 2015–2016, 2016–2017), U.S. Forest Service personnel collected hair samples from fishers from the KR and SP study areas during capture and radio-collaring operations (mid-October to late February). We followed capture and monitoring techniques described by Green et al. [74]. During processing, we measured body length (cm), weight (kg), tail length (cm), teeth length (mm), and reproductive status through teat condition and testicular size. We shaved an area on the neck for blood sampling, and this shaved hair was collected for analysis; the same area was shaved on each fisher to standardize the area of hair collection [66]. Fishers were fitted with a VHF radio-collar weighing 31 g (Holohil model MI-2M, Holohil Systems Ltd., Carp, Ontario, Canada) with a handmade breakaway [40]. We injected each fisher with a Passive Integrated Transponder (Biomark, Boise, ID) for future identification and tracking [74]. At capture, we measured body mass (kg) and body length (cm) from the tip of the nose along the back of the spine to the base of the tail to determine BMI. We captured and handled animals under authorization of the U.S. Forest Service with permits from California Department of Fish and Wildlife (Permit SC-2730) with Institutional Animal Care and Use Committee review and approval from the University of California-Davis (IACUC #18022), and followed capture and handling protocols recommended by the American Society of Mammalogists [75].

### Sample extractions and radioimmunoassay

Protocol for hair processing and extraction followed [10, 20]. The mass of hair samples ranged from 35 to 200 mg; sample mass was used to adjust for final cortisol sample concentration of each individual sample. We washed hair samples three times for 3 minutes each with HPLC-grade methanol to remove any dirt and waste. We air-dried and weighed the hair samples, then placed the sample in a 25-ml chamber with a 7-mm steel grinding ball (Restch ball mill, Verder Scientific, Germany). The hair sample was ground for 20 min at 30 Hz. We collected and assayed a subsample of each of three washes to assure no hormone was lost during washing. We then weighed the ground hair and transferred the hair to a 5-ml Eppendorf tube. We added 3 ml of methanol to the ground hair samples and they were placed on a vortexer for a 24-hr period to facilitate extraction of the hormone from the ground hair to the methanol. We then centrifuged the ground hair samples for 10 min at 914 g, and 1.5 ml of supernatant was removed and placed in a clean glass tube. This volume was chosen so as to not remove any of the hair during separation of the supernatant. The supernatant was then dried under a stream of nitrogen and re-suspended in 0.2 ml of PBS buffer. We assayed samples using a radioimmunoassay in duplicate for cortisol 3 antibody [16]. Species validation was performed to test for non-specific binding, binding interference, and parallelism prior to running all samples. The assay included a 10-point standard curve run in triplicate with known values of cortisol. The intra-assay variation was 4.0%. All assayed samples fell within the standard curve for the assay. We performed a species validation for the radioimmunoassay and antibody prior to study samples and found good parallelism with an R^2^ = 0.986, and no significant interference using tests with known high and low spikes of cortisol which yielded an average recovery of 94.8%.

### Home range estimates

Cortisol in hair indicates an accumulation of physiological responses over months depending on growth and molt cycles [64], suggesting hair can be used as a measure of chronic stress. Fishers molt and replace their fur from September through early November [39], thus accumulation of cortisol from the body into the hair occurs during this molting period and active hair growth [14, 66]. Hair collected in February will still represent cortisol accumulation between September through early November when the hair is growing. Whether the hair follicles of fishers stop growth by early November is unknown [76], but 93% (59 of 64) of the hair samples were collected after November 1. A comprehensive meta-analysis of the literature supported hair cortisol analysis as a sensitive measure of central hypothalamic-pituitary-adrenal axis function and a biomarker of persistent or ongoing stressors [77].

To associate the level of cortisol in a hair sample with the time when cortisol was accumulated in the hair, we used the annual home range prior to when the hair sample was collected; all hair samples were collected during trapping operations from mid-October to late February. Home ranges from the year before the sample was collected were associated with the hair samples because the location the individual inhabited the year before would influence the individuals’ chronic stress levels. Essentially, animals were initially captured and radio-collared, then recaptured approximately a year later and a hair sample collected which represented an accumulation of cortisol from the prior year. Therefore, the previous annual home range was used as a conservative measure of space use and reflected exposure to activities and landscape changes within their home range during the previous year.

We determined annual home ranges using radio-telemetry locations, rest sites, aerial locations, and den sites [74]. Rest sites were defined as a single structure in which an individual was located (i.e., tree, snag, burrow). Rest areas were defined as an area (within 50 m) in which an individual was located, but could not be narrowed down to one specific structure [74]. Locations (triangulations, rest sites, and rest areas) of female fishers were obtained approximately every 3 days, with rest site locations and rest areas were collected opportunistically when searching an area. Den locations were added into the data set once for every 3 days a female was at a den; this was based on how often female fishers are typically relocated, and these locations were subsampled as to not skew the home range estimators towards these den sites. The telemetry error for each location type varied but were similar among fishers and therefore any bias was weighted equally among individuals. We analyzed females and males separately since males use a larger area and do not use dens, while females have smaller home ranges and restrict their movements when raising young; males do not assist with raising young [78]). We used program R (adehabitatHR package; version 3.3.0) in R studio (version 1.0.136, [79]) and ArcGIS 10.5 (ESRI, Redlands, CA, USA) to determine annual home ranges with 30%, 60%, and 95% isopleths [80]. These three spatial scales were used to determine whether individuals experienced different stressors in the core (30%) of their home ranges/areas of use versus larger areas (60%) and the entirety of their home ranges/areas of use (95%). We examined these three scales because the levels of anthropogenic influences varied among the three home range scales [81]. We calculated area-observation curves for each fisher and determined 25–30 points were needed to estimate the home ranges of females. We defined the areas the males inhabited as “areas of use” because the area-observation curves for males never reached an asymptote.

### Anthropogenic modifications

We obtained data layers of known and mapped silvicultural treatments on the study areas for each year from the Forest Service Activity Tracking System (FACTS) database (https://data.fs.usda.gov/geodata/edw/datasets.php); data layer had a spatial resolution of 1 m. Management activities included (a) thinning of natural fuels, (b) commercial thinning, (c) full planting without concurrent site preparation, (d) fill-in re-planting without concurrent site preparation, (e) individual tree release and weeding, and (f) pre-commercial thinning of individual or selected trees. We calculated the area of each activity for each female home range or male area of use at the three kernel sizes, and converted to the proportion (m^2^ of activity/km^2^ of home range or area of use) of each activity in each home range during each year. We used m^2^ instead of km^2^ because some management activities were very small. The U.S. Forest Service provided locations of buildings and roads in each study area; spatial resolution of 1 m. Using GIS, we determined building density (# buildings/km^2^) and road density (m of roads/km^2^) within each female’s home range, or each male’s area of use at all three kernel sizes. We used meters for road length instead of km because some roads were <1 km. Road density consisted of paved, dirt, and decommissioned roads; the available data layer combined all of these roads into one category.

### Habitat characteristics

In addition to anthropogenic modifications, we examined two habitat characteristics (canopy cover, vegetation type) on both areas for each year as a measure of habitat quality. We obtained canopy cover and vegetation data from the LANDFIRE database (U.S. Forest Service; U.S. Department of Interior) which has a native spatial resolution of 30 m. We delineated eight vegetative types: conifer forest, hardwood forest, mixed hardwood-conifer forest, granite, water, developed land, shrubland, and sparse cover. We measured the percent of each habitat type available in each home range or area of use at each kernel estimator. Prior research in the study areas characterized dense canopy as ≥60% canopy cover [40]. Therefore, canopy cover was divided into 3 classes: ≥60% (dense), 41–59% (moderate), and ≤40% (low). Our goal in dividing canopy cover into these three categories was to determine whether any of these canopy cover levels influenced cortisol, and which levels may be more tolerated by fishers. In the future, these canopy classes could be used to determine a canopy cover threshold that fishers prefer. We calculated the percentage of each canopy class for each home range or area of use at each kernel estimator.

### Tree mortality

We obtained tree mortality data from the U.S. Department of Agriculture Remote Sensing Lab (www.fs.usda.gov/detail/catreemortality/toolkit/?cid=fseprd498067) and intersected this data layer with the home ranges and areas of use using ArcGIS; data was measured as the number of dead trees per hectare. We reported tree mortality as the number of dead trees per acre. Data on levels of tree mortality were available for the years 2015 and 2016 only; measurable tree mortality was not recorded in 2014.

### Components of fitness

We examined how levels of cortisol influenced three fitness parameters (body condition, survival, reproduction). For allometric body condition, we calculated a body mass index (BMI) as the ratio of body mass (kg)/body length (cm) [82]. We used linear regression to examine the relationship between cortisol and BMI. We determined survival intervals for the time when the individual was radio-collared until September 15 of the year following their capture. We used September 15 as the end-point for the interval each year because this was when new hair began to grow [39]. However, for animals captured during the 2016–2017 trapping season, we used May 1, 2017, when project monitoring ended. We calculated male and female survival rates and 95% confidence intervals for each year using the program MICROMORT [83]. To examine the influence of cortisol on survival rates, we divided animals into three classes of cortisol (low: <0.12 pg/mg, medium: 0.121–0.189 pg/mg, high: >0.190 pg/mg); classes were chosen by dividing the animals into thirds based on the distribution of cortisol values. We performed a z-test to determine differences in survival rates among years and classes of cortisol levels.

Denning season for females and their kits occurs in April-June. Once we identified the natal den tree via telemetry, we placed remote cameras to monitor the tree until the female moved her kits to the next den tree; we used these photos to determine litter size [74]. We climbed den trees after the female moved her kits to document mortality or abandonment. To investigate the influence of cortisol on reproduction, we examined the relationship between cortisol and litter size during the denning season before and after the sample was collected. This analysis examined (a) whether the number of kits a female produced was associated with her cortisol level, or (b) whether her cortisol level was associated with the number of kits produced the following year. As we were examining chronic stress instead of acute stress, cortisol accumulating in the hair during molting season could be influenced by the previous reproductive season, or could influence the following reproductive season. We used analysis of variance to examine the relationship between cortisol and the number of kits produced by a female for the year before and the year after the cortisol sample was collected.

### Statistical analysis

We used R (version 3.3.0) in R studio (version 1.0.136; [79]) for statistical analysis. When the data did not conform to a normal distribution, we would log or square root transform the data. We used generalized linear mixed models (GLMM) with normal distribution followed by Akaike Information Criterion (AIC_c_) model selection [84] due to our small sample sizes to determine which measures of anthropogenic modifications, habitat, tree mortality, and the additive and interactive combination of these factors, influenced cortisol levels in female and male fishers at each of the home range estimators (30, 60, 95% isopleths). Due to different life histories and space use patterns, we analyzed female and male fishers separately. Because we often sampled fishers from more than one year, we included ‘individual’ as a random effect. Data on tree mortality was only available for 2015 and 2016, thus we could only use these years for model selection. To reduce the number of models, we analyzed models in a sequential process or phased-modeling approach [85–89]. The sequential process was used to create a more rigorous and thoughtful approach to the analysis instead of incorporating every variable into the final model. In the first phase, we analyzed canopy cover, management activity, and vegetation type as univariate models and all possible additive and interactive combinations of these covariates. With the sequential approach, the variables from the top model in each group (canopy cover, vegetation type, management activity) were then moved to the global model. In the next phase, we then added road density, building density, and tree mortality to the global model for final model analysis. Any variables that were correlated (r > 0.6) through the cor.test function in program R were not included in the same model. The global model included all possible additive and interactive combinations. We did not include study area or year in the models because we were examining all these variables within each individual fishers’ home range on a yearly basis (i.e., these categorical variables were presented by the continuous variables for each individual fisher). Models with a weight ≥0.1 were considered significant and model weight aided in model inference. For statistical tests, we reported the *P*-values and considered *P* < 0.10 as an indicator of significance.

## Results

**Fig 1:**
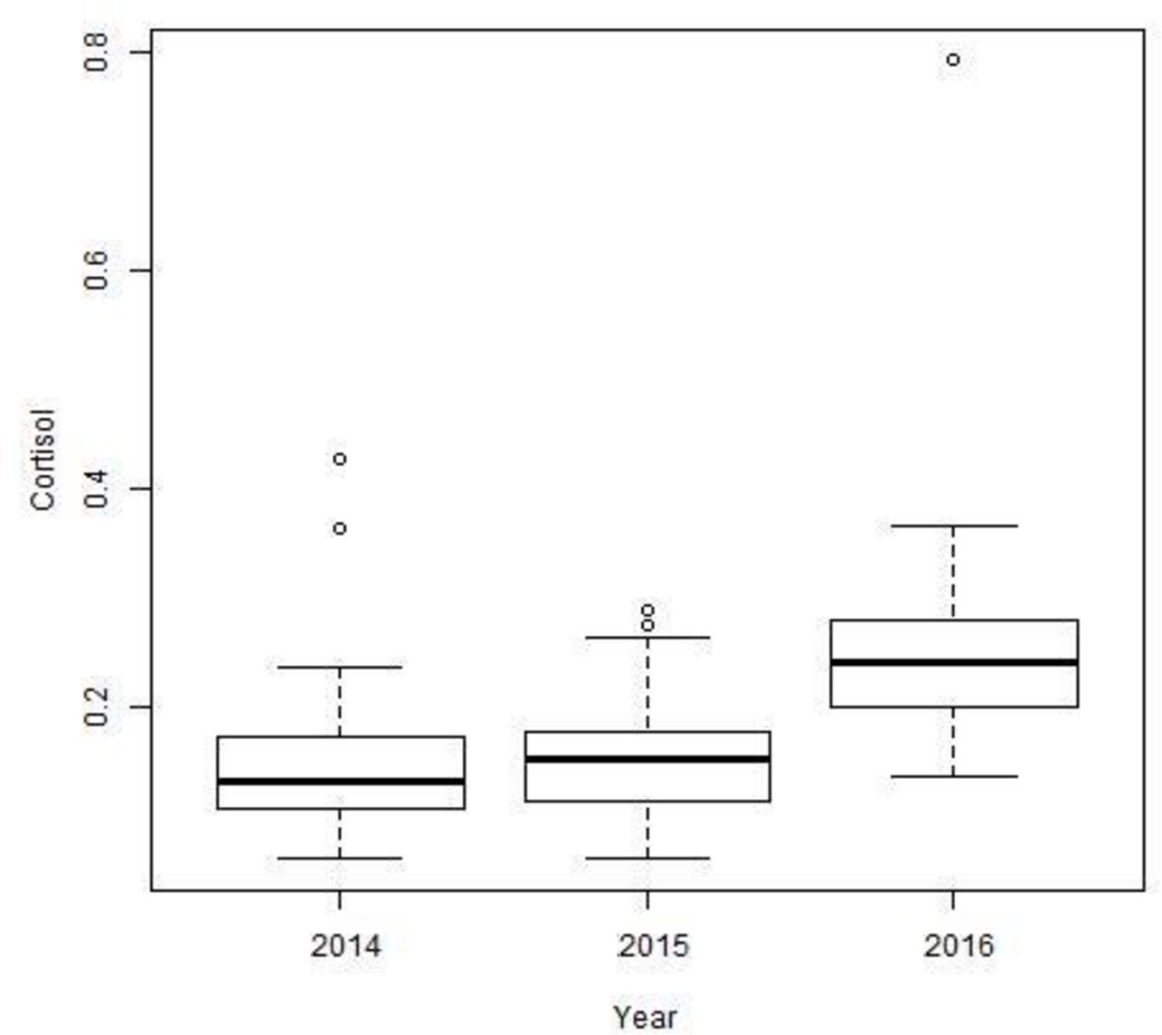
Cortisol levels (pg/mg) for female and male fishers combined in 2014, 2015, and 2016, Sierra National Forest, California, USA.

In 2014–2016, we captured 23 male (KR: 10, SP: 13) and 41 female (KR: 22, SP: 19) fishers. We captured some individuals more than once contributing to 32 hair samples obtained from males (KR: 19, SP: 13) and 68 hair samples from females (KR: 44, SP: 24). During each trapping season, each individual was only captured and measured once; animals recaptured in the same trapping season were immediately released without sampling. Home range size for female fishers averaged 1.92 ± 1.88 (SD), 5.92 ± 5.58, and 21.82 ± 18.16 km^2^, for the 30%, 60%, and 95% isopleths, respectively. Areas of use for male fishers averaged 9.28 ± 7.30, 26.33 ± 19.65, and 88.56 ± 54.79 km^2^, respectively. The percent of the home range impacted by management activities was very small for both female and male fishers (Table 1). Cortisol levels averaged 0.182 ± 0.103 (SD) pg/mg (range: 0.0609–0.7922 pg/mg) for females, and 0.163 ± 0.066 pg/mg (range: 0.0878–0.2863 pg/mg) for males (t_131_ = -1.3384, *P* = 0.183). Cortisol levels of both males and females were relatively similar in the first two years, but increased in the third year (Fig 1; F_2, 130_ = 25.96, *P* < 0.001). Cortisol levels averaged 0.188 ± 0.099 pg/mg (range: 0.0899–0.7922 pg/mg) in the Kings River study site, and 0.146 ± 0.056 pg/mg (range: 0.0609–0.2877 pg/mg) in the Sugar Pine study site.

**Table 1:** Metrics (mean ± SD) of building density, road density, tree mortality, and percent of the home range affected by management activities in the 95% kernel home ranges of female and male fishers, Sierra National Forest, California, 2014–2016.

Variable	Females	Males
Building density (#/km^2^)	0.54±0.79	2.38±2.85
Road density (m of road/km^2^)	2,052.79±633.99	2,000.89±601.97
Tree mortality (# dead trees/acre)	20.48±21.47	16.93±12.76
% thinning of natural fuels	0.0005±0.0029	0.0003±0.0033
% commercial thinning	0.0054±0.0342	0.0002±0.0004
% full planting without concurrent site preparation	0.0038±0.0141	0.0001±0.0002
% fill-in re-planting without concurrent site preparation	0	0.0011±0.0021
% individual tree release and weeding	0.0075±0.0316	0.0068±0.0085
% precommercial thinning of individual or selected trees	0.0229±0.0699	0.0046±0.0068

### Influence of anthropogenic modifications, habitat, and tree mortality on cortisol levels in female fishers

**Fig 2:**
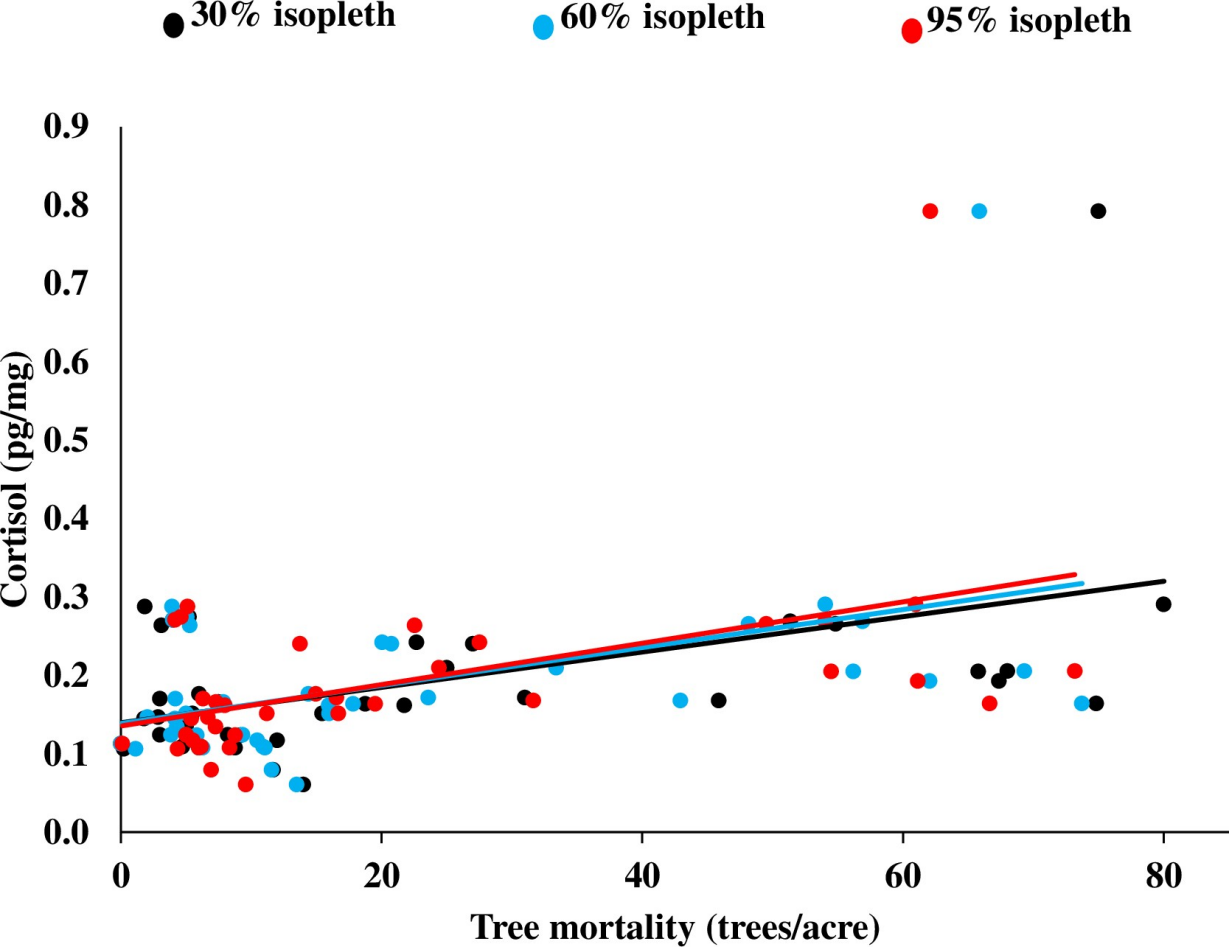
Relationship between cortisol (pg/mg) in hair and the amount of tree mortality (trees/acre) in the 30%, 60%, and 95% kernel home ranges for female fishers, Sierra National Forest, California, USA, 2014–2016.

We collected 68 hair samples from 41 females during 2014–2016, but only used 40 hair samples collected from 30 females during 2015–2016 to examine model selection in the years that had tree mortality data. After removing models with correlated variables (different variables were correlated at different spatial scales for each sex), we constructed 36 models examining the influence of various anthropogenic modifications, habitat, and tree mortality within the 30% isopleth on cortisol levels of females. For females, a model containing only tree mortality had the highest influence on cortisol (Table 2) with increasing cortisol related to increasing tree mortality (*r* = 0.49; Fig 2). A second model containing tree mortality and percent developed habitat added an additional 10.5% model weight, with the 2 top-ranked models carrying 35.4% of the model weight (Table 2). Although both models were over the threshold of weight ≥0.1, tree mortality was the only significant variable with all other variables considered uninformative (variable coefficients had *P*-values > 0.1; [90]. Tree mortality was present in all of the 10 top-performing models, with these 10 models containing tree mortality having a combined model weight of 80.0%. The one apparent outlier was a female with high cortisol (Fig 2), but there was no apparent error in lab analysis, thus we retained this data point. However, removing this possible outlier produced similar model results with the 2 top-ranked models containing tree mortality as the only significant variable having a combined weight of 24.7%. Tree mortality was still in all of the 10 top-performing models with these 10 models having a combined weight of 69.9%.

**Table 2:** Models with weight ≥0.1 examining metrics of anthropogenic modifications, habitat, and tree mortality in the 30, 60, and 95% kernel home range of individual female fishers and the influence on hair cortisol levels, Sierra Nevada Mountains, California, 2014–2016.

Model	ΔAICc	Weight	*P*-value for variables
**30% kernel**			
Tree mortality	0.0	0.248	0.0013
Tree mortality + % developed	1.7	0.105	Tree mortality: 0.0017
			% developed: 0.4031
**60% kernel**			
Tree mortality	0.0	0.210	0.0024
Tree mortality + % hardwood_conifer	0.3	0.184	Tree mortality: 0.0103
			% hardwood_conifer: 0.1555
**95% kernel**			
Tree mortality	0.0	0.538	0.0011
Tree mortality + Pre-commercial thinning of individual or selected trees	2.0	0.197	Tree mortality: 0.0017
			Pre-commercial thinning of individual or selected trees: 0.5114
Tree mortality + Building density	2.4	0.159	Tree mortality: 0.0013
			Building density: 0.8428

For the 60% isopleth, we constructed 48 models after removing models with correlated variables. The top-ranked model influencing cortisol in females included only tree mortality (Table 2) with cortisol increasing as tree mortality increased (*r* = 0.47; Fig 2). The second-ranked model included tree mortality and percent hardwood-conifer, with the 2 top-ranked models carrying 39.4% of model weight (Table 2). Although these models were over the threshold of weight ≥0.1, again only tree mortality was significant with all other variables considered uninformative [90]. Tree mortality was present in all top-10 performing models, with these 10 models containing tree mortality having a combined weight of 77.0%. Similar to the results for the 30% isopleth, removing the one possible outlier produced similar model results with tree mortality in 9 of the 10 top-performing models with these 9 models having a combined weight of 62.2%.

For the 95% isopleth, we constructed 19 models after removing models with correlated variables. The top-ranked model again contained only tree mortality as influencing cortisol of females (Table 2) with cortisol increasing with increased tree mortality (*r* = 0.50; Fig 2). Two additional models that included tree mortality, pre-commercial thinning of individual or selected trees, and building density with model weights >0.1 weight had a combined model weight of 89.5% (Table 2). Although these models had weights ≥0.1, tree mortality was significant in the 3 top-ranked models and all other variables were considered uninformative [90]. Tree mortality was present in only 4 of the 10 top-performing models, but these 4 models containing tree mortality having a combined weight of 94.5%. Again, removing the one possible outlier produced models with tree mortality in 6 of the 10 top-performing models with these 6 models having a combined weight of 86.0%.

### Influence of anthropogenic modifications, habitat, and tree mortality on cortisol levels in male fishers

**Fig 3:**
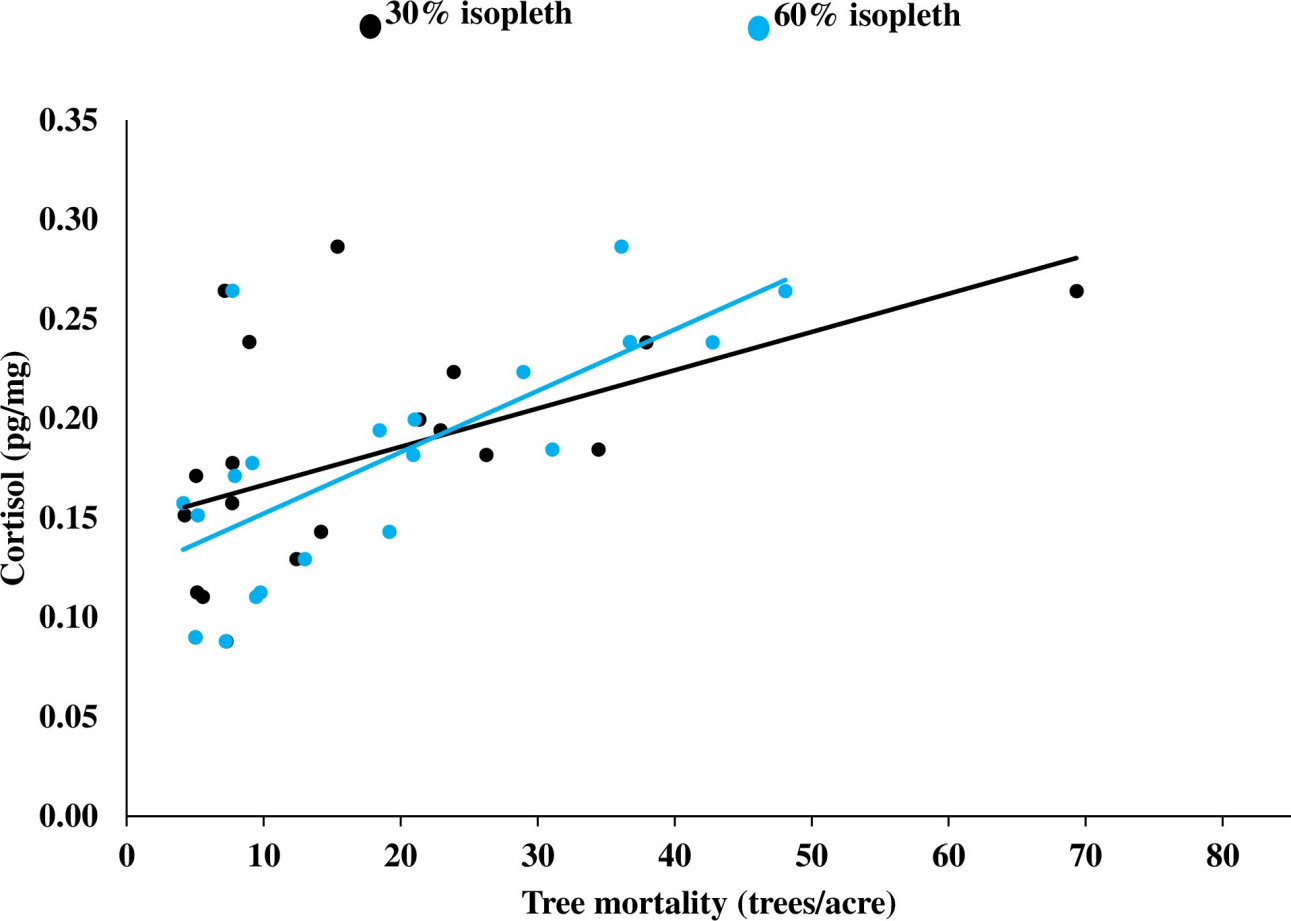
Relationship between cortisol (pg/mg) in hair and the amount of tree mortality (trees/acre) in the 30 and 60% kernel areas of use for male fishers, Sierra National Forest, California, USA, 2014–2016.

We used 32 hair samples collected from 20 males during 2015–2016 to develop 48 models (after removing models with correlated variables) examining the influence of anthropogenic modifications, habitat, and tree mortality within the 30% isopleth area of use on cortisol levels of male fishers. The top-ranked model for males contained tree mortality, percent sparse cover, and pre-commercial thinning of individual or selected tress (Table 3) with cortisol increasing in areas with increased tree mortality (*r* = 0.52; Fig 3), as well as areas of use with increased sparse cover and increasing per-commercial thinning of individual or selected trees. Two other models containing tree mortality, percent low canopy cover, and amount of pre-commercial thinning of individual or selected trees had model weights >0.1 with these 3 top-ranked models having a combined model weight of 55.0% (Table 3). Although these models had model weight ≥0.1, tree mortality and the amount of pre-commercial thinning were the only significant variables with all other variables considered uninformative [90]. Tree mortality was present in all of the 10 top-performing models, with these 10 models having a combined model weight of 81.8%.

**Table 3:** Models with weight ≥0.1 examining metrics of anthropogenic modifications, habitat, and tree mortality in the 30, 60, and 95% kernel areas of use for individual male fishers and the influence on hair cortisol levels, Sierra Nevada Mountains, California, 2014–2016.

Model	ΔAICc	Weight	*P*-value for variables
**30% kernel**			
Tree mortality + % sparse + pre-commercial thinning of individual or selected trees	0.0	0.219	Tree mortality: 0.0063
			% sparse: 0.0876
			Pre-commercial thinning of individual or selected trees: 0.0181
Tree mortality + pre-commercial thinning of individual or selected trees	0.1	0.204	Tree mortality: 0.0083
			Pre-commercial thinning or individual or selected trees: 0.0252
Tree mortality + % low canopy cover + pre-commercial thinning of individual or selected trees	1.1	0.127	Tree mortality: 0.0056
			% low canopy cover: 0.1495
			Pre-commercial thinning of individual or selected trees: 0.0338
**60% kernel**			
Tree mortality	0.0	0.264	0.0004
Tree mortality + pre-commercial thinning of individual or selected trees	1.2	0.147	Tree mortality: 0.0016
			Pre-commercial thinning of individual or selected trees: 0.2001
Tree mortality + % sparse	1.2	0.144	Tree mortality: 0.0009
			% sparse: 0.2045
Tree mortality + % low canopy cover	1.9	0.102	Tree mortality: 0.0001
			% low canopy cover: 0.3067
**95% kernel**			
Tree mortality + road density	0.0	0.338	Tree mortality: 0.0069
			Road density: 0.0131
Tree mortality + road density + building density	0.6	0.250	Tree mortality: 0.0028
			Road density: 0.0113
			Building density: 0.1259

For the 60% isopleth, we constructed 40 models after removing models with correlated variables and found the top-ranked model contained only tree mortality (Table 3) with cortisol increasing as tree mortality increased (*r* = 0.72; Fig 3). Three other models containing tree mortality, the amount of pre-commercial thinning of individual or selected trees, percent sparse cover, and percent low canopy cover had model weights >0.1, with these 4 top-ranked models carrying 65.6% of the model weight (Table 3). Although these models had model weights ≥0.1, only tree mortality was significant with all other variables considered uninformative [90]. Tree mortality was present in all of the 10 top-performing models with these 10 models having a combined model weight of 90.1%.

**Fig 4:**
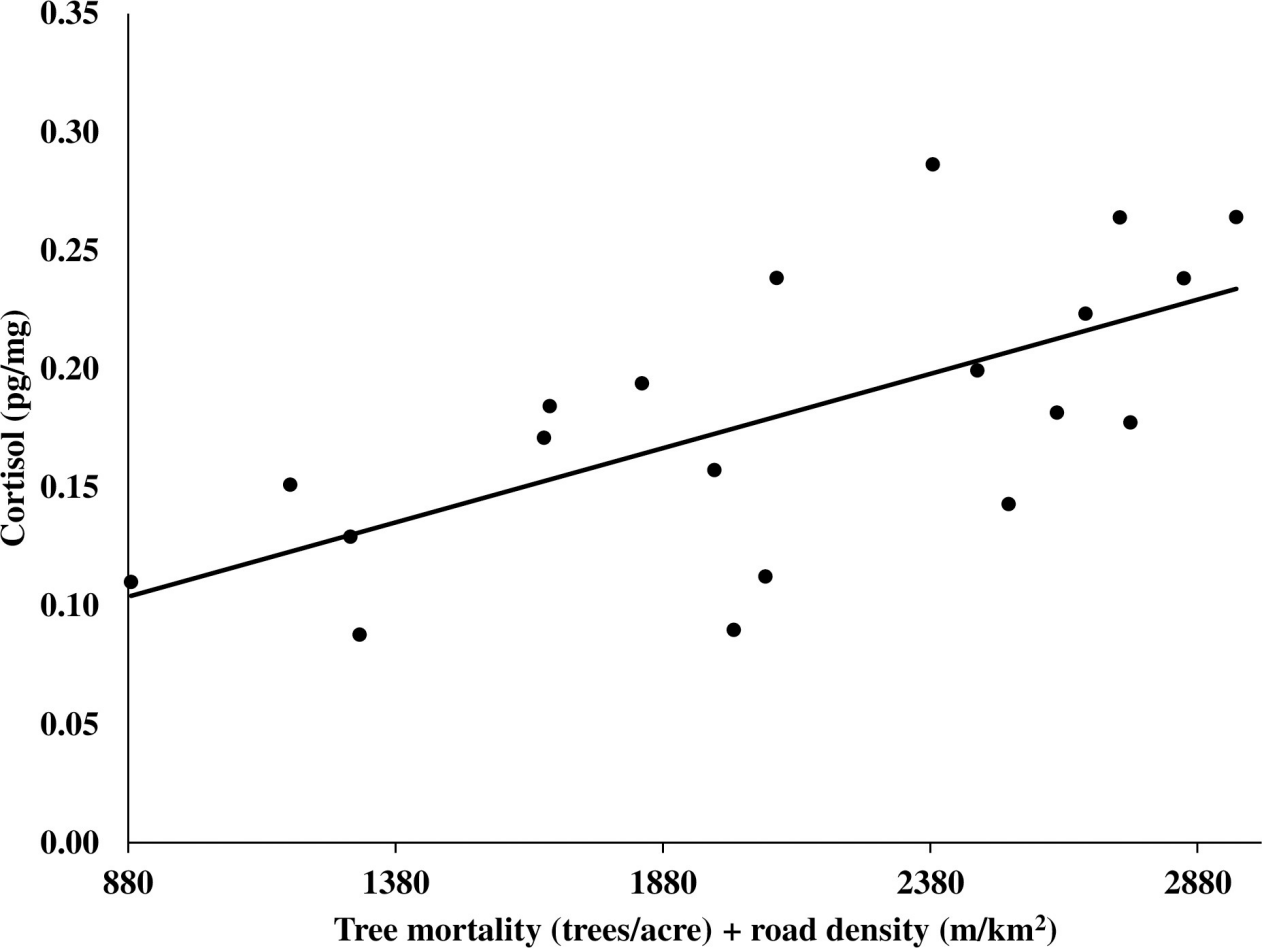
Relationship between cortisol (pg/mg) in hair and the amount of tree mortality (trees/acre) and road density (m/km^2^) in the 95% kernel areas of use for male fishers, Sierra National Forest, California, USA, 2014–2016.

For the 95% isopleth, we constructed 26 models after removing models with correlated variables. We found the top-ranked model contained the variables of tree mortality and road density (Table 3) with cortisol increasing as tree mortality and road density increased (Fig 4). A second-ranked model containing tree mortality, road density, and building density added an additional 25% of model weight, with the 2 top-ranked models having a combined model weight of 58.8% (Table 3). Both tree mortality and road density were significant variables with all other variables considered uninformative [90]. Tree mortality was present in 7 of the 10 top-ranked models with these 7 models having a combined model weight of 85.1%.

### Influence of cortisol level on fitness

We examined the relationship between BMI and cortisol concentrations using samples from the 68 females and 32 males collected during 2014–2016, and found no relationship between cortisol and individual BMI estimates (*F*_1, 117_ = 0.356, *P* = 0.552). There was no significant correlation between individual cortisol levels and BMI for females (*r* = 0.131, *P* > 0.40) or males (*r* = -0.078, *P* > 0.50).

We examined 37 females to determine if the number of kits produced influenced the subsequent cortisol level of an individual female fisher after giving birth. We found no difference in cortisol concentrations among females after they produced 0, 1, 2, or 3 kits (*F*_3, 33_ = 0.354, *P* = 0.786). We also examined if cortisol influenced the subsequent litter size for 54 females where we were able to obtain kit counts and found no difference in cortisol prior to giving birth and whether they subsequently produced 0, 1, 2, or 3 kits (*F*_3, 50_ = 1.251, *P* = 0.301). The average litter size across the two study areas was 1.57 kits per female [74] with 25%, 33%, 32%, and 10% of females having litters of 0, 1, 2, and 3 kits, respectively.

**Fig 5:**
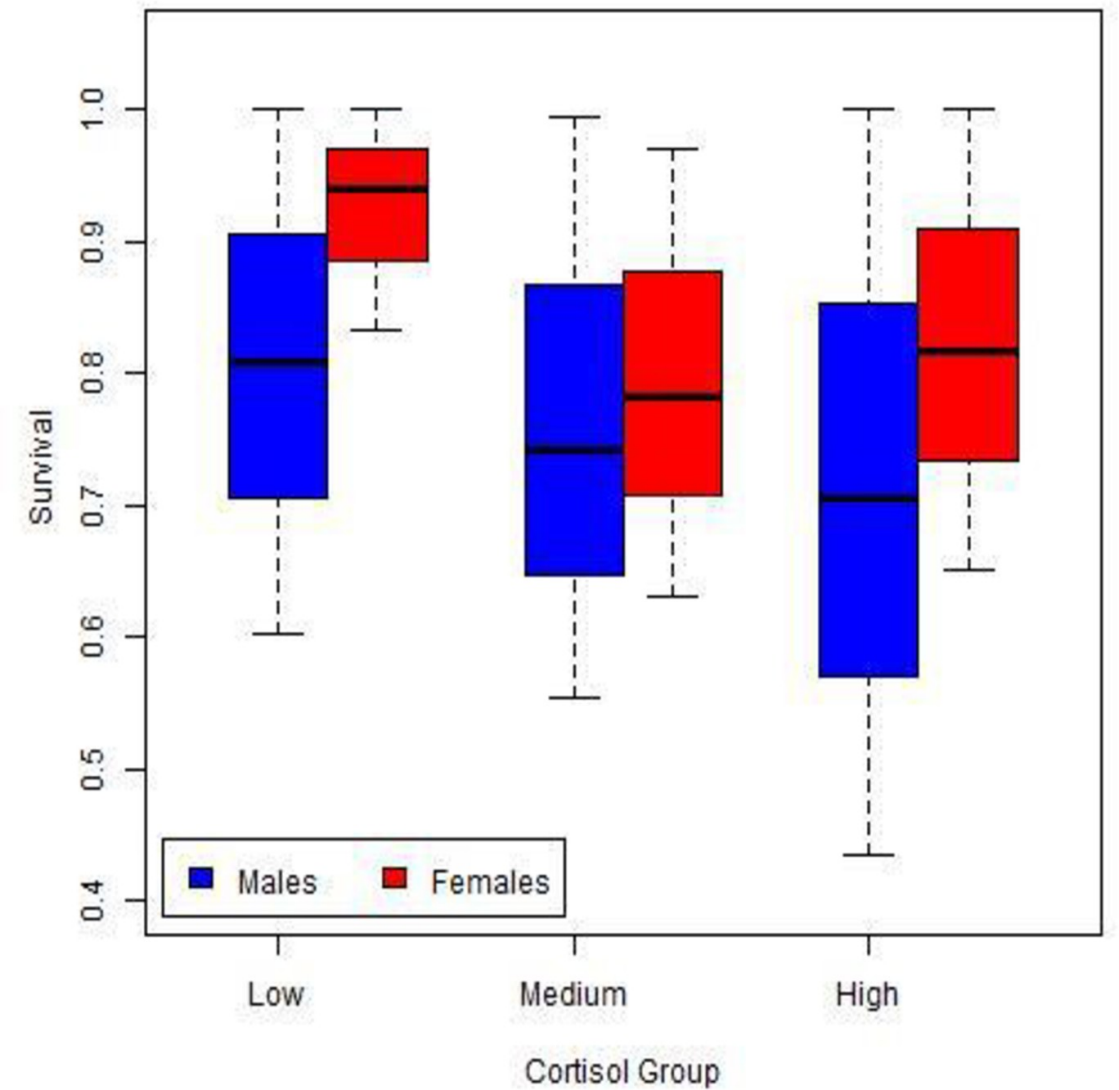
Annual survival rates of male and female fishers in relation to their level of cortisol, Sierra National Forest, California, USA, 2014–2016. Cortisol classes were low (<0.12 pg/mg), medium (0.121–0.189 pg/mg), and high (>0.190 pg/mg); error bars represent 95% confidence intervals.

Using 17,664 radio-days for females, annual survival rates showed an increasing trend over the 3 years, but there was no difference among years (all z-tests had *P* > 0.26). When we examined the relationship between cortisol levels and annual survival rates (Fig 5), females with low cortisol concentrations had the highest annual survival rates (0.94) compared to females with medium (0.78; z = 1.56, *P* = 0.06) or high (0.81; z = 1.44, *P* = 0.07) cortisol concentration. Annual survival rates for females with medium and high levels of cortisol did not differ (z = 0.27, *P* = 0.39).

Using 9,373 radio-days for males, annual survival rates generally increased over time, but there was no difference amongst years (all z-tests had *P* > 0.30). Among the 3 classes of cortisol levels, males exhibited a similar trend as females with males with low cortisol levels having higher survival (0.81), and medium and high cortisol levels having lower survival, 0.74 and 0.71, respectively. However, there was no statistical difference in annual survival rates among males with low, medium, and high levels of cortisol (all z-tests had *P* > 0.17; Fig 5).

## Discussion

This is the first study documenting a link between increased tree mortality and increased cortisol levels in fishers. For female and male fishers, tree mortality was the most important variable influencing cortisol levels at all spatial scales, such that increased cortisol was associated with increased tree mortality. Our results showed tree mortality, as related to the effects of the 4-year drought, is the principle driver creating a “landscape of stress” for fishers in the Sierra Nevada Mountains. Tree mortality could influence fishers by reducing thermoregulatory cover, loss of escape terrain, or decreased prey base. Loss of canopy cover (escape terrain) may lead to increased predation risk from other carnivores. Decreased canopy cover due to tree mortality may increase the outside temperature or reproductive den temperature due to lack of shade. Kilpatrick and Rego [78] reported fishers selected rest sites in part due to thermoregulation. If the amount of late-successional forest continues to decrease with increased tree fall [91], these animals will also have fewer trees to use for shelter and dens. With the loss of trees and decreased cone production, small mammals will likely decline during subsequent years. Numerous studies have shown prey declines can lead to changes in reproduction and reduced body condition in carnivores [92, 93]. If the small mammal prey base declines, this isolated fisher population could be adversely impacted.

Levels of tree mortality have increased in the Sierras due to the recent 4-year drought and increases in mountain pine beetles attacking the already struggling trees [94] with high tree mortality (>80% of stands) observed throughout the area. Tree mortality due to drought and pine beetle infestation will likely continue to increase as climate change increases the severity of droughts and insect infestations [52, 53]. Although the winter of 2016–2017 experienced greater than average levels of precipitation, the high levels of tree mortality were irreversible [94]. If tree mortality continues to increase with the resulting increase in tree fall, the amount of late-successional forest that fishers prefer will also continue to decrease [91].

Within the 95% isopleth for male fishers, we found the combined effect of tree mortality and road density had the greatest influence on cortisol. The additive effect of road density likely reflects the life history of male fishers. Mountain lions (*Puma concolor*), which prey on fishers [56, 95], frequently use roads to hunt, and given the male fishers’ widespread movement, increased road density may increase encounter rates with mountain lions, which could lead to increased cortisol in males and potentially decrease survival.

Pre-commercial thinning of individual or selected trees was the only management action contained in the top-ranked models suggesting fishers in the study areas were not negatively influenced by most management actions. This supports other studies reporting animals can adapt and may even benefit from anthropogenic modifications [96]. Levine [97] showed environmental stressors that are controllable or predictable may not be perceived as stressful. Anthropogenic modifications can usually be avoided compared to widespread tree mortality. The areas impacted by human activities were very selective in placement and small in area, while tree mortality occurred across the landscape. One study [41] showed fishers may tolerate small amounts of management on the landscape, and another study [44] documented resident fishers responded to management activities within their territory by travelling around impacted areas but did not alter their overall home range. In contrast, tree mortality was severe in both areas, with mortality of Ponderosa pines ≥80% [94]. The effects of tree mortality were unavoidable amongst individual fishers, whereas anthropogenic modifications which were low in density and occurred in small patches may allow the animals to concentrate their activity to areas devoid of human activities and anthropogenic modifications.

Average cortisol levels for fishers sampled in the KR area were generally higher than in the SP area likely due to differences in tree mortality in the two areas. In the SP area, males had an average of 10.45 dead trees/acre and females averaged 7.5 dead trees/acre. In contrast, in the KR area, males had an average of 19.71 dead trees/acre, and females averaged 26.04 dead trees/acre. The large difference in tree mortality between the two areas is likely influencing the level of cortisol levels in the individual fishers across these two study populations. Topographic and microclimatic differences between these sites likely influenced different rates of pine beetle infection and subsequent tree mortality.

Most importantly, we found female fishers with low cortisol levels had higher annual survival rates than females with medium and high cortisol levels. Although females with high cortisol had slightly higher survival than females with medium cortisol levels, these two groups did not differ in survival. Decreased survival in females could greatly influence the future of this small, isolated population. Although not statistically significant, the trend among males was similar to the females with decreasing survival rates as cortisol levels increased; lack of significance was likely due to small sample size. This is the first study linking a physiological stress response to changes on the landscape to subsequent fitness (i.e., survival rate) among female fishers. Similarly, Rakotoniaina et al. [98] reported that high levels of hair cortisol was associated with reduced survival probabilities in grey mouse lemurs (*Microcebus murinus*). In boreal woodland caribou (*Rangifer tarandus caribou*), individuals with more of their home range having been logged best explained the variance in hair cortisol concentrations [99]. Lavergne et al. [100] reported a relationship between stress profiles and risk avoidance behavior among snowshoe hares (*Lepus americanus*). Linking anthropogenic changes in an animal’s home range to physiological stress responses and subsequent fitness consequences is important for future management and conservation.

## Conclusion

Tree mortality, in response to the 4-year drought, is creating a “landscape of stress” within this fisher population. This study is the first to show a link between tree mortality and physiological stress in fishers. Even more significant was the link between increased cortisol and decreased female survival. During this study, cortisol increased over the 3 years which could cause fitness to decline if tree mortality continues to increase and decreases the amount of late-successional forest [40]. Climate change is continuing to increase the average temperatures of this region [101]. If cortisol levels continue to increase, we may begin to see greater impacts of physiological stress on the fitness and health of individuals in this population. With anticipated increases in drought severity due to climate change, the landscape of stress will likely continue to persist and expand. By understanding how climate change is influencing this sensitive species, forest managers may be able to conserve the late-successional forest this fisher population occupies. Additionally, the methods of determining the physiological stress response to landscape change has important applications to other forest carnivores in this region. Two other mesocarnivores (ringtails, *Bassariscus astutus*, and pine marten, *Martes americana*) in this region could also be equally impacted by changes in forest structure.

## References

[1] Brown JS, Kotler BP. Hazardous duty pay and the foraging cost of predation. Ecol Letters. 2004; 7:999–1014. 10.1111/j.1461-0248.2004.00661.x

[2] Schmitz OJ. Scaling from plot experiments to landscapes: studying grasshoppers to inform forest ecosystem management. Oecologia. 2005; 145:224–233. doi: 10.1007/s00442-005-0063-y 15891842

[3] Preisser EL, Orrock JL, Schmitz OJ. Predator hunting mode and habitat domain alter nonconsumptive effects in predator–prey interactions. Ecology. 2007; 88:2744–2751. doi: 10.1890/07-0260.1 18051642

[4] Laundré JW, Hernández L, Ripple WJ. The landscape of fear: ecological implications of being afraid. Open Ecol J. 2010; 3:1–7. 10.2174/1874213001003030001

[5] Kohl MT, Stahler DR, Metz MC, Forester JD, Kauffman MJ, Varley N, et al. Diel predator activity drives a dynamic landscape of fear. Ecol Monogr. 2018; 88:638–652. 10.1002/ecm.1313

[6] Cunningham CX, Johnson CN, Hollings T, Kreger K, Jones ME. Trophic rewilding establishes a landscape of fear: Tasmanian devil introduction increases risk-sensitive foraging in a key prey species. Ecography. 2019; 42:2053–2059. 10.1111/ecog.04635

[7] Suraci JP, Clinchy M, Zanette LY, Wilmers CC. Fear of humans as apex predators has landscape-scale impacts from mountain lions to mice. Ecol Letters. 2019; 22:1578–1586. doi: 10.1111/ele.13344 31313436

[8] Beehner JC, Bergman TJ. The next step for stress research in primates: to identify relationships between glucocorticoid secretion and fitness. Horm Behav. 2017; 91:68–83. doi: 10.1016/j.yhbeh.2017.03.003 28284709

[9] Davenport MD, Tiefenbacher S, Lutz CK, Novak MA, Meyer JS. Analysis of endogenous cortisol concentrations in the hair of rhesus macaques. Gen Comp Endocrinol. 2006; 147:255–261. doi: 10.1016/j.ygcen.2006.01.005 16483573

[10] Macbeth BJ, Cattet MRL, Stenhouse GB, Gibeau ML, Janz DM. Hair cortisol concentration as a noninvasive measure of long-term stress in free-ranging grizzly bears (*Ursus arctos*): considerations with implications for other wildlife. Can J Zool. 2010; 88:935–949. 10.1139/z10-057

[11] Myers MJ, Litz B, Atkinson S. The effects of age, sex, season and geographic region on circulating serum cortisol concentrations in threatened and endangered Steller sea lions (*Eumetopias jubatus*). Gen Comp Endocrinol. 2010; 165:72–77. doi: 10.1016/j.ygcen.2009.06.006 19524580

[12] Dantzer B, Fletcher QE, Boonstra R, Sheriff MJ. Measures of physiological stress: a transparent or opaque window into the status, management, and conservation of species? Conserv Physiol. 2014; 2. doi: 10.1093/conphys/cou023 27293644 PMC4732472

[13] Boonstra R. Reality as the leading cause of stress: rethinking the impact of chronic stress in nature. Functional Ecol. 2013; 27:11–23. 10.1111/1365-2435.12008

[14] Heimbürge S, Kanitz E, Otten W. The use of hair cortisol for the assessment of stress in animals. Gen Comp Endocrinol. 2019; 270:10–17. doi: 10.1016/j.ygcen.2018.09.016 30287191

[15] Romero LM. Physiological stress in ecology: lessons from biomedical research. Trends Ecol Evol. 2004; 19:249–255. doi: 10.1016/j.tree.2004.03.008 16701264

[16] French SS, DeNardo DF, Greives TJ, Strand CR, Demas GE. Human disturbance alters endocrine and immune responses in the Galapagos marine iguana (*Amblyrhynchus cristatus*). Horm Behav. 2010; 58:792–799. doi: 10.1016/j.yhbeh.2010.08.001 20708010 PMC2982938

[17] Fourie NH, Turner TR, Brown JL, Pampush JD, Lorenz JG, Bernstein RM. Variation in vervet (*Chlorocebus aethiops*) hair cortisol concentrations reflects ecological disturbance by humans. Primates. 2015; 56:365–373. doi: 10.1007/s10329-015-0486-y 26318176

[18] Neuman-Lee LA, Brodie ED, Hansen T, French SS. To stress or not to stress: Physiological responses to tetrodotoxin in resistant garter snakes vary by sex. Comp Biochem Physiol A: Molecular Integrative Physiol. 2017a; 209:34–40. 10.1016/j.cbpa.2015.11.017 28380330

[19] Mislan P, Derocher AE, St. Louis VL, Richardson E, Lunn NJ, Janz DM. Assessing stress in Western Hudson Bay polar bears using hair cortisol concentration as a biomarker. Ecol Indicators. 2016; 71:47–54. 10.1016/j.ecolind.2016.06.034

[20] Neuman-Lee LA, Terletzky PA, Atwood TC, Gese EM, Smith GD, Greenfield S, et al. Demographic and temporal variations in immunity and condition of polar bears (*Ursus maritimus*) from the southern Beaufort Sea. J Exp Zool A: Ecol Integrative Physiol. 2017b; 327:333–346. 10.1002/jez.2112 29356384

[21] Sheriff MJ, Dantzer B, Delehanty B, Palm R, Boonstra R. Measuring stress in wildlife: techniques for quantifying glucocorticoids. Oecologia. 2011; 166:869–887. doi: 10.1007/s00442-011-1943-y 21344254

[22] Wingfield JC. Ecological processes and the ecology of stress: the impacts of abiotic environmental factors. Functional Ecol. 2013; 27:37–44. 10.1111/1365-2435.12039

[23] Tilbrook AJ, Turner AI, Clarke IJ. Effects of stress on reproduction in non-rodent mammals: the role of glucocorticoids and sex differences. Rev Reproduction. 2000; 5:105–113. doi: 10.1530/ror.0.0050105 10864855

[24] Love OP, Williams TD. The adaptive value of stress-induced phenotypes: effects of maternally derived corticosterone on sex-biased investment, cost of reproduction, and maternal fitness. Amer Nat. 2008; 172:135–149. 10.1086/590959 18793091

[25] Ebensperger LA, Tapia D, Ramirez-Estrada J, Leon C, Soto-Gamboa M, Hayes LD. Fecal cortisol levels predict breeding but not survival of females in short-lived rodent, *Octodon degus*. Gen Comp Endocrinol. 2013; 186:164–171. doi: 10.1016/j.ygcen.2013.02.044 23524002

[26] Schneiderman N, Ironson G, Siegel SD. Stress and health: psychological, behavioral, and biological determinants. Ann Rev Clinical Psychol. 2005; 1:607–628. 10.1146/annurev.clinpsy.1.102803.144141 17716101 PMC2568977

[27] Glaser R, Kiecolt-Glaser JK. Stress-induced immune dysfunction: implications for health. Nature Rev Immunol. 2005; 5:243–251. doi: 10.1038/nri1571 15738954

[28] Bonier F, Martin PR, Moore IT, Wingfield JC. Do baseline glucocorticoids predict fitness? Trends Ecol Evol. 2009; 24:634–642. doi: 10.1016/j.tree.2009.04.013 19679371

[29] Dantzer B, Newman AEM, Boonstra R, Palme R, Boutin S, Humphries MM, et al. Density triggers maternal hormones that increase adaptive offspring growth in a wild mammal. Science. 2013; 340:1215–1217. doi: 10.1126/science.1235765 23599265

[30] Romero M, Dickens M, Cyr N. The reactive scope model- a new model integrating homeostasis, allostasis, and stress. Horm Behav. 2009; 55:375–389. doi: 10.1016/j.yhbeh.2008.12.009 19470371

[31] Gaillard JM, Festa-Bianchet M, Yoccoz NG, Loison A, Toigo C. Temporal variation in fitness components and population dynamics of large herbivores. Ann Rev Ecol Evol Syst. 2000; 31:367–393. 10.1146/annurev.ecolsys.31.1.367

[32] Wingfield JC. Comparative endocrinology, environment and global change. Gen Comp Endocrinol. 2008; 157:207–216. doi: 10.1016/j.ygcen.2008.04.017 18558405

[33] Rangel-Negrin A, Alfaro JL, Valdez RA, Romano MC, Serio-Silva JC. Stress in Yucatan spider monkeys: effects of environmental conditions on fecal cortisol levels in wild and captive populations. Anim Conserv. 2009; 12:496–502. 10.1111/j.1469-1795.2009.00280.x

[34] Hoegh-Guldberg O, Hughes L, McIntyre S, Lindenmayer DB, Parmesan C, Possingham HP, et al. Assisted colonization and rapid climate change. Science. 2008; 321:345–346. doi: 10.1126/science.1157897 18635780

[35] Touma C, Palme R. Measuring fecal glucocorticoid metabolites in mammals and birds: the importance of validation. Annals New York Acad Sci. 2005; 1046:54–74. doi: 10.1196/annals.1343.006 16055843

[36] Gunderson AR, Stillman JH. Plasticity in thermal tolerance has limited potential to buffer ectotherms from global warming. Proc Royal Soc B: Biol Sci. 2015; 282:20150401. doi: 10.1098/rspb.2015.0401 25994676 PMC4455808

[37] Koolhaas JM, Korte SM, DeBoer SF, Van Der Vegt BJ, Van Reenen CG, Hopster H, et al. Coping styles in animals: current status in behavior and stress-physiology. Neuroscience Biobehav Rev. 1999; 23:925–935. doi: 10.1016/s0149-7634(99)00026-3 10580307

[38] Möstl E, Palme R. Hormones as indicators of stress. Domestic Anim Endocrinol. 2002; 23:67–74. doi: 10.1016/s0739-7240(02)00146-7 12142227

[39] Douglas CW, Strickland MA. 1987. Fisher. In: Novak M, Baker JA, Obbard ME, Malloch B, editors. Wild furbearer management and conservation in North America. Toronto: Ontario Ministry of Natural Resources, Canada; 1987. pp. 511–530.

[40] Thompson CM, Zielinski WJ, Purcell KL. Evaluating management risks using landscape trajectory analysis: A case study of California fisher. J Wildl Manage. 2011; 75:1164–1176. 10.1002/jwmg.159

[41] Zielinski WJ, Thompson CM, Purcell KL, Garner JD. An assessment of fisher (*Pekania pennanti*) tolerance to forest management intensity on the landscape. For Ecol Manage. 2013; 310:821–826. 10.1016/j.foreco.2013.09.028

[42] Schempf PF, White M. Status of six furbearer populations in the mountains of northern California. Museum of Vertebrate Zoology, University of California, Berkeley. 1977.

[43] Zielinski WJ, Truex RL, Schlexar FV, Campbell LA, Carroll C. Historical and contemporary distribution of carnivores in forests of the Sierra Nevada, California, USA. J Biogeography. 2005; 32:1385–1407. 10.1111/j.1365-2699.2005.01234.x

[44] Sweitzer RA, Furnas BJ, Barrett RH, Purcell KL, Thompson CM. Landscape fuel reduction, forest fire, and biophysical linkages to local habitat use and local persistence of fishers (*Pekania pennanti*) in Sierra Nevada mixed-conifer forests. For Ecol Manage. 2016; 361:208–225. 10.1016/j.foreco.2015.11.026

[45] Lewis JC, Zielinski WJ. Historical harvest and incidental capture of fishers in California. Northwest Sci. 1996; 70:291–297.

[46] Spencer W, Rustigian-Romsos H, Strittholt J, Scheller R, Zielinski W, Truex R. Using occupancy and population models to assess habitat conservation opportunities for an isolated carnivore population. Biol Conserv. 2011; 144:788–803. 10.1016/j.biocon.2010.10.027

[47] Tucker JM, Allendorf FW, Truex RL, Schwartz MK. Sex-biased dispersal and spatial heterogeneity affect landscape resistance to gene flow in fisher. Ecosphere. 2017; 8:e01839. 10.1002/ecs2.1839

[48] Knaus BJ, Cronn R, Liston A, Pilgrim K, Schwartz MK. Mitochondrial genome sequences illuminate maternal lineages of conservation concern in a rare carnivore. BMC Ecology. 2011; 11:10. doi: 10.1186/1472-6785-11-10 21507265 PMC3108907

[49] Tucker JM, Schwartz MK, Truex RL, Pilgrim KL, Allendorf FW. Historical and contemporary DNA indicate fisher decline and isolation occurred prior to the European settlement of California. PLoS ONE. 2012; 7:e52803. doi: 10.1371/journal.pone.0052803 23300783 PMC3530519

[50] U.S. Fish and Wildlife Service. 2014. Endangered and threatened wildlife and plants; threatened species status for West Coast Distinct Population Segment. Federal Register 79:60419–60425.

[51] Bart RR, Tague CC, Moritz MA. Effect of tree-to-shrub type conversion in lower montane forests of the Sierra Nevada (USA) on streamflow. PLoS ONE. 2016; 11:e0161805. doi: 10.1371/journal.pone.0161805 27575592 PMC5004902

[52] Dale VH, Joyce LA, McNulty S, Neilson RP, Ayres MP, Flannigan MD, et al. Climate change and forest disturbances: climate change can affect forests by altering the frequency, intensity, duration, and timing of fire, drought, introduced species, insect and pathogen outbreaks, hurricanes, windstorms, ice storms, or landslides. BioScience. 2001; 51:723–734. https://doi-org.dist.lib.usu.edu/10.1641/0006-3568(2001)051[0723:ccafd]2.0.co;2

[53] Allen CD, Macalady AK, Chenchouni H, Bachelet D, McDowell N, Vennetier M, et al. A global overview of drought and heat-induced tree mortality reveals emerging climate change risks for forests. For Ecol Manage. 2010; 259:660–684. 10.5772/56277

[54] Franklin JP, Fites-Kaufman J. Assessment of late-successional forests of the Sierra Nevada. Sierra Nevada Ecosystem Project, University of California, Davis, California; 1996.

[55] Heilman GE, Strittholt JR, Slosser NC, Dellasala DA. Forest fragmentation of the conterminous United States: assessing forest intactness through road density and spatial characteristics: forest fragmentation can be measured and monitored in a powerful new way by combining remote sensing, geographic information systems, and analytical software. BioScience. 2002; 52:411–422. 10.1641/0006-3568(2002)052[0411:ffotcu]2.0.co;2

[56] Wengert GM, Gabriel MW, Matthews SM, Higley JM, Sweitzer RA, Thompson CM, et al. Using DNA to describe and quantify interspecific killing of fishers in California. J Wildl Manage. 2014; 78:603–611. 10.1002/jwmg.698

[57] Sweitzer RA, Popescu VD, Barrett RH, Purcell KL, Thompson CM. Reproduction, abundance, and population growth for a fisher (*Pekania pennanti*) population in the Sierra National Forest, California. J Mammal. 2015; 96:772–790. 10.1093/jmammal/gyv083

[58] Purcell KL, Mazzoni AK, Mori SR, Boroski BB. Resting structures and resting habitat of fishers in the southern Sierra Nevada, California. For Ecol Manage. 2009; 258:2696–2706. 10.1016/j.foreco.2009.09.041

[59] Allen-Diaz B, Standiford R, Jackson RD. Oak woodlands and forests. In: Barbour MG, Keeler-Wolf T, Schoenherr AA, editors. Terrestrial Vegetation of California, 3rd edition. Berkley: University of California Press; 2007. pp. 313–338. 10.1525/california/9780520249554.001.0001

[60] Keeley JE, Davis FW. 2007. Chaparral. In: Barbour MG, KeelerWolf T, Schoenherr AA, editors. Terrestrial Vegetation of California, 3rd edition. Berkeley: University of California Press. pp. 339–366. 10.1525/california/9780520249554.001.0001

[61] Fettig CJ, Mortenson LA, Bulaon BM, Foulk PB. Tree mortality following drought in the central and southern Sierra Nevada, California, U.S. For Ecol Manage. 2019; 432:164–178. 10.1016/j.foreco.2018.09.006

[62] Dettmer AM, Novak MA, Suomi SJ, Meyer JS. Physiological and behavioral adaptation to relocation stress in differentially reared rhesus monkeys: hair cortisol as a biomarker for anxiety-related responses. Psychoneuroendocrinol. 2012; 37:191–199. doi: 10.1016/j.psyneuen.2011.06.003 21715101 PMC3196800

[63] Meyer JS, Novak MA. Minireview: Hair cortisol: A novel biomarker of hypothalamic-pituitary-adrenocortical activity. Endocrinol. 2012; 153:4120–4127. doi: 10.1210/en.2012-1226 22778226 PMC3423616

[64] Tallo-Parra O, Lopez-Bejar M, Carbajal A, Monclús L, Manteca X, Devant M. Acute ACTH-induced elevations of circulating cortisol do not affect hair cortisol concentrations in calves. Gen Comp Endocrinol. 2016; 240:138–142. doi: 10.1016/j.ygcen.2016.10.007 27777047

[65] McCormick SD, Romero LM. Conservation endocrinology. BioScience. 2017; 67:429–442. 10.1093/biosci/bix026

[66] Lavergne SG, Peers MJL, Mastromonaco G, Majchrzak YN, Nair A, Boutin S, et al. Hair cortisol as a reliable indicator of stress physiology in the snowshoe hare: influence of body region, sex, season, and predator-prey population dynamics. Gen Comp Endocrinol. 2020; 294:113471. doi: 10.1016/j.ygcen.2020.113471 32234297

[67] Sharpley CF, Kauter KG, McFarlane JR. 2010. An investigation of hair cortisol concentration across body sites and within hair shaft. Clin Med Insights Endocrinol Diabetes. 2010; 3:17–23. doi: 10.4137/cmed.s4465 22879783 PMC3411542

[68] Keckeis K, Lepschy M, Schöpper H, Moser L, Troxler J, Palme R. Hair cortisol: a parameter of chronic stress? Insights from a radiometabolism study in guinea pigs. J Comp Physiol B. 2012; 182(7):985–996. doi: 10.1007/s00360-012-0674-7 22592890

[69] Koren L, Bryan H, Matas D, Tinman S, Fahlman Å, Whiteside D, et al. Towards the validation of endogenous steroid testing in wildlife hair. J Appl Ecol. 2019; 56:547–561.

[70] Greff MJE, Levine JM, Abuzgaia AM, Elzagallaai AA, Rieder MJ, van Uum SHM. Hair cortisol analysis: an update on methodological considerations and clinical applications. Clinical Biochem. 2019; 63:1–9. doi: 10.1016/j.clinbiochem.2018.09.010 30261181

[71] Macbeth BJ, Cattet MR, Obbard ME, Middel K, Janz DM. Evaluation of hair cortisol concentration as a biomarker of long‐term stress in free‐ranging polar bears. Wildl Soc Bull. 2012; 36:747–758. 10.1002/wsb.219

[72] Bechshøft TO, Sonne C, Rigét FF, Letcher RJ, Novak MA, Henchey E, et al. Polar bear stress hormone cortisol fluctuates with the North Atlantic Oscillation climate index. Polar Biol. 2013; 36:1525–1529. 10.1007/s00300-013-1364-y

[73] Palme R. Non-invasive measurement of glucocorticoids: Advances and problems. Physio Behav. 2019; 199:229–243. doi: 10.1016/j.physbeh.2018.11.021 30468744

[74] Green RE, Purcell KL, Thompson CM, Kelt DA, Wittmer HU. Reproductive parameters of the fisher in the southern Sierra Nevada, California. J Mammal. 2018; 99:537–553. 10.26686/wgtn.12510047

[75] Sikes RS, Animal Care and Use Committee of the American Society of Mammalogists. 2016 Guidelines of the American Society of Mammalogists for the use of wild mammals in research and education. J Mammal. 2016; 97:663–688. doi: 10.1093/jmammal/gyw078 29692469 PMC5909806

[76] Geyfman M, Plikus MV, Treffeisen E, Andersen B, Paus R. Resting no more: re-defining telogen, the maintenance stage of the hair growth cycle. Biol Rev. 2015; 90:1179–1196. doi: 10.1111/brv.12151 25410793 PMC4437968

[77] Kalliokoski O, Jellestad FK, Murison R. A systematic review of studies utilizing hair glucocorticoids as a measure of stress suggests the marker is more appropriate for quantifying short-term stressors. Scientific Rep. 2019; 9:11997. doi: 10.1038/s41598-019-48517-2 31427664 PMC6701156

[78] Kilpatrick HJ, Rego PW. Influence of season, sex, and site availability on fisher (*Martes pennanti*) rest-site selection in the central hardwood forest. Can J Zool. 1994; 72:1416–1419. 10.1139/z94-187

[79] R Development Core Team. 2016. R: a language and environment for statistical computing. Foundation for Statistical Computing, Vienna, Austria.

[80] Worton BJ. Kernel methods for estimating the utilization distribution in home-range studies. Ecology. 1989; 70:164–168. 10.2307/1938423

[81] Kordosky JR, Gese EM, Thompson CM, Terletzky PA, Purcell KL, Schneiderman JD. Landscape use by fishers: core areas differ in habitat than the entire home range. Can J Zool. 2021; in press.

[82] Jakob EM, Marshall SD, Uetz GW. Estimating fitness: a comparison of body condition indices. Oikos. 1996; 77:61–67. 10.2307/3545585

[83] Heisey DM, Fuller TK. Evaluation of survival and cause-specific mortality rates using telemetry data. J Wildl Manage. 1985; 49:668–674. 10.2307/3801692

[84] Burnham KP, Anderson DR. Model selection and inference: a practical information-theoretic approach. New York: Springer-Verlag; 2002.

[85] Nichols JD, Hines JE, Blums P. Tests for senescent decline in annual survival probabilities of common pochards, *Aythya ferina*. Ecology. 1997; 78:1009–1018. 10.1890/0012-9658(1997)078[1009:tfsdia]2.0.co;2

[86] Franklin AB, Gutiérrez RJ, Nichols JD, Seamans ME, White GC, Zimmerman GS, et al. Population dynamics of the California spotted owl (*Strix occidentalis occidentalis*): a meta-analysis. Ornithol Monogr. 2004; 54:1–54. 10.2307/40166799

[87] Blakesley JA, Seamans ME, Conner MM, Franklin AB, White GC, Gutiérrez RJ, et al. Population dynamics of spotted owls in the Sierra Nevada, California. Wildl Monogr. 2010; 174:1–36. 10.2193/2008-475

[88] Symonds MRE, Moussalli A. A brief guide to model selection, multimodel inference and model averaging in behavioural ecology using Akaike’s information criterion. Behav Ecol Sociobiol. 2011; 65:13–21. 10.1007/s00265-010-1037-6

[89] Anderson-Cook CM, Morzinski J, Blecker KD. Statistical model selection for better prediction and discovering science mechanisms that affect reliability. Systems. 2015; 3:109–132. 10.3390/systems3030109

[90] Arnold TW. Uninformative parameters and model selection using Akaike’s Information Criterion. J Wildl Manage. 2010; 74:1175–1178. 10.1111/j.1937-2817.2010.tb01236.x

[91] Van Mantgem PJ, Stephson NL, Byrne JC, Daniels LD, Franklin JF, Fule PZ, et al. Widespread increase of tree mortality rates in the western United States. Science. 2009; 323:521–524. doi: 10.1126/science.1165000 19164752

[92] Ward RMP, Krebs CJ. Behavioural responses of lynx to declining snowshoe hare abundance. Can J Zool. 1985; 63:2817–2824. 10.1139/z85-421

[93] O’Donoghue M, Boutin S, Krebs CJ, Hofer EJ. Numerical responses of coyotes and lynx to the snowshoe hare cycle. Oikos. 1997; 80:150–162. 10.2307/3546526

[94] Young DJN, Stevens JT, Earles JM, Moore J, Ellis A, Jirka AL, et al. Long-term climate and competition explain forest mortality patterns under extreme drought. Ecol Letters. 2017; 20:78–86. doi: 10.1111/ele.12711 28000432

[95] Gabriel MW, Woods LW, Wengert GM, Stephenson N, Highley JM, Thompson C, et al. Patterns of natural and human-caused mortality factors of a rare forest carnivore, the fisher (*Pekania pennanti*) in California. PLoS ONE. 2015; 10:e0140640. doi: 10.1371/journal.pone.0140640 26536481 PMC4633177

[96] Arnould JPY, Monk J, Ierodiaconou D, Hindell MA, Semmens J, Hoskins AJ, et al. Use of anthropogenic sea floor structures by Australian fur seals: potential positive ecological impacts of marine industrial development? PLoS ONE. 2015; 10:e0130581. doi: 10.1371/journal.pone.0130581 26132329 PMC4488539

[97] Levine S. Influence of psychological variables on the activity of the hypothalamic-pituitary-adrenal axis. European J Pharmacol. 2000; 405:149–160. doi: 10.1016/s0014-2999(00)00548-3 11033322

[98] Rakotoniaina JH, Kappeler PM, Kaesler E, Hämäläinen AM, Kirschbaum C, Kraus SG. Hair cortisol concentrations correlate negatively with survival in a wild primate population. BMC Ecology. 2017; 17:1–13. doi: 10.1186/s12898-016-0111-y 28859635 PMC5579956

[99] Ewacha MVA, Roth JD, Anderson WG, Brannen DC, Dupont DLJ. Disturbance and chronic levels of cortisol in boreal woodland caribou. J Wildl Manage. 2017; 81:1266–1275. 10.1002/jwmg.21288

[100] Lavergne SG, Smith K, Kenney A, Krebs CJ, Palme R, Boonstra R. Physiology and behavior of juvenile snowshoe hares at the start of the 10-year cycle. Anim Behav. 2019; 157:141–152. 10.1016/j.anbehav.2019.09.003

[101] Stewart IT, Cayan DR, Dettinger MD. Changes in snowmelt runoff timing in western North America under a ‘business as usual’ climate change scenario. Climatic Change. 2004; 62:217–232. 10.1023/b:clim.0000013702.22656.e8

